# Utilization and predictors of long acting reversible contraceptive methods among reproductive age women in Hawassa city, South Ethiopia: a community based mixed methods

**DOI:** 10.1186/s40834-020-00112-x

**Published:** 2020-07-06

**Authors:** Aklilu Tilahun, Amanuel Yoseph, Mesay Hailu Dangisso

**Affiliations:** 1 Adare General Hospital, Maternal and Child Health service case team, Hawassa, Ethiopia; 2 School of Public Health, College of Medicine and Health Sciences, Hawassa University, Hawassa, Ethiopia

**Keywords:** Reproductive age women, Long acting reversible contraceptive methods, Utilization, Hawassa city and Ethiopia

## Abstract

**Background:**

Long acting reversible contraceptive methods are highly effective, safe and provide uninterrupted protection to women for 3 to 12 years, yet are little used in the Ethiopia. Assessment of the utilization and predictors of long acting reversible contraceptive methods assist health planners to prioritize promotion strategies, and is a fundamental step for intervention. Therefore, this study aimed to assess the utilization and predictors of long acting reversible contraceptives among reproductive age women in Hawassa city, South Ethiopia; 2019.

**Methods:**

A community-based cross-sectional study was conducted using a mixed method among the sample of 660 reproductive age women in Hawassa city, South Ethiopia from January 1–30, 2019. We have used a systematic and purposive sampling technique to select the study participants. A structured interview-administrated questionnaire and focus group discussion were used to collect the data. The data were entered using Epi data version 3.1 and analyzed using SPSS version 20. Chi-square (X^2^) test was used to determine the overall association between explanatory and outcome variables. The variables were entered into the multivariable model using the backward stepwise regression approach. Bi-variable and multivariable logistic regression analyses were conducted. The qualitative data were analyzed using a manual thematic analysis technique.

**Results:**

The overall utilization of long acting reversible contraceptive methods was 22% (95% CI = 19.50–25.50%). Among this, 17.5 and 4.5% of women utilized the implants and IUCD, respectively. Good knowledge (AOR = 4.0; 95% CI = 1.66–9.60; *P* = 0.001) and positive attitude (AOR = 7.9; 95% CI = 3.84–16.10; *P* = 0.001) of women about LARC methods were positively associated with utilization of LARC methods. The odds of utilizing LARC methods increased 8.2 times for women who have no desire to have a child (AOR = 8.2, 95% CI = 3.13–21.30) as compared to those who have the desire to have a child. The discussion of women about LARC methods with providers (AOR = 4.1; 95% CI = 1.24–5.24) and husbands (AOR = 2.7; 95% CI =1.02–7.20) were positively associated with utilization of LARC methods. These findings were supported by the individual, institutional and socio-cultural qualitative findings.

**Conclusions:**

The utilization of LARC methods far below the national target (22 V 40%) in the study area. Good knowledge and positive attitude about LARC methods, no desire to have a child, discussion with husbands and providers were major predictors of the LARC methods utilization. Increasing knowledge and positive attitude of the women about LARC methods using various methods of health education should be considered.

## Background

Contraceptive methods are classified as modern and traditional methods. Modern methods include female sterilization, male sterilization, the intrauterine contraceptive device (IUCD), implants, injectable, the pill, male condoms, female condoms, emergency contraception, standard day’s method (SDM), and lactation amenorrhea method (LAM). Methods such as rhythm, withdrawal, and folk method are grouped as traditional [1].

Hormonal contraceptive implants are a reversible long acting contraceptive which release a progestin hormone in the body. It can give a continuous protection for 3 to 5 years. The IUCD is also a reversible long acting contraceptive which is a small device placed in the uterus to prevent pregnancy [2].

Contraceptive use was estimated to prevent 218 millions of unintended pregnancies in developing countries. It also prevented 55 millions of unplanned births, 138 millions of abortions (of which 40 million are unsafe) and 25 million miscarriages. Moreover, it prevents 118,000 maternal deaths and the loss of 240,000 healthy years from women’s lives each year [3].

Globally, the prevalence of unintended pregnancies and unsafe abortion is high. Its implication in terms of public health is alarming. According to WHO report, it was estimated that 282 and 40 million women had unintended pregnancies and unsafe abortion, 30 million women were suffering from the most severe form of unsafe abortion complication and 5–10 million annual deaths were among 15–49 years in 2016 [4].

The economic cost of unintended pregnancies and unsafe abortion are substantial which leads to total loss of 10% disability-adjusted life years [5]. It also resulted in increases in fertility by 15% [3]. Consequently, unintended pregnancies and unsafe abortion might end up with 10% losses in productivity [4, 5].

Low utilization of the modern contraceptive methods is a great challenge in most low resource settings, no exception in Ethiopia. Among the six top underutilized countries (India, Nigeria, Pakistan, Afghanistan and the DRC) in which almost half of worldwide maternal mortality happens, an estimated maternal mortality ratio of Ethiopia is 420 per 100,000 live births [1, 2].

The huge number of maternal death, specifically in low and middle income countries has been because of low level of LARC methods utilization. The low level utilization of LARC methods compounded by the extremely low ANC, skilled delivery and PNC utilization which are the major predictors for the high maternal deaths during the last two decades [6].

In addition, different predictors have been found to be associated with the utilization of LARC methods and can be grouped as socio-demographic, behavioral, institutional and service related factors [7].

In spite of the presence of many modern contraceptive methods options such as pills, injectable, implants, IUCD and sterilization, low utilization of LARC methods are a wide-spread problem in Ethiopia [2].

The Ethiopian Federal Ministry of Health developed health sector transformation plan and promised to achieve 40% utilization of the LARC methods by 2020 [8]. However, a study conducted in Adigrat town of Tigray region, Ethiopia shows that utilization of LARC methods is 28.7% [9]. Injectable and pills are the commonest and widely utilized contraceptive methods throughout country (Ethiopia) [2].

Modern contraceptive methods service utilization has been among the most relevant interventions strategy to reduce maternal morbidity and mortality [10]. Due to this evidence, Ethiopia has given a special consideration to it in the last three decades [8]. Moreover, modern contraceptive methods especially focus on LARC methods, which are among the six priority areas in the reproductive health strategy of the country to enhance maternal health [11].

In spite of the fact that modern contraceptive methods service utilization is very important for further improvement of maternal health, limited information exists on the utilization and predictors of LARC methods among reproductive age women in Hawasa city, South Ethiopia. In addition, information about LARC methods is also important because it is one of the targets for Ethiopian GTP 2 in 2012/2020 and the preparation for the third Growth and Transformation Plan in Ethiopia are fast approaching. Moreover, assessing the current level of utilization and identifying the predictors of LARC methods is important to guide public health planners, policymakers and implementers to plan and design appropriate intervention strategies in order to enhance LARC methods utilization. Therefore, the main aim of this study was to assess utilization and predicators of LARC methods among reproductive age women in Hawassa city, South Ethiopia, 2019.

## Methods

### Study area

The study was carried out in Hawassa city, South Ethiopia. The Hawassa city is located 275 km from Addis Ababa, the capital of Ethiopia. According to the central statistical agency report of Ethiopia, the total population of the city was estimated to be 359,358 (49% males and 51% females). The city has consisted of 8 sub-cities and 32 Kebeles (smallest administrative unit of Ethiopia). The health service coverage of the city was 94%. There is only one-government referral hospital, one general hospital, one primary hospital, 10 health centers and 32 health post. The city also consists of 12 private hospitals, 30 private clinics and 45 pharmacies. According to the health department report, the distribution of underutilization of LARC methods affects almost all Kebeles of the city. Trade is the main source of income in the Hawassa city; inhabitants of the rural sub-cities mainly produce enset (*Enset ventricosum*), cereals, cash crops (khat, coffee) and livestock (Hawassa City Administration: Annual health sector reports on health and other health related issues, unpublished).

### Study design and population

A community-based cross-sectional study was carried out using quantitative and qualitative methods in Hawassa city from January 1–30; 2019. The source and study population were all women in reproductive age group who were non-pregnant and all systematically selected women in reproductive age group who were non-pregnant and resided in the Hawassa city for 6 months, respectively. Pregnant women were excluded from the study.

### Sample size determination

For the first objective, the sample size was calculated by using a single population proportion formula in consideration of the following assumptions. The utilization/proportion (p) of long acting reversible contraceptive methods from the previous study done in Adigrat town, Northern Ethiopia was 28.7% [9], 95% confidence level, 5% margin of error. As a two-stage sampling technique was used to select the study participants, a design effect of 2 was considered and a 10% non-response rate was added to the calculated sample size. Therefore, the final sample size was 692. Similarly, the sample size (n) for the second objective was calculated by using the EPI-info TM 7 statistical package with the inputs of: 95% confidence level, 80% power, 1:1 ratio between exposed and unexposed and adjusted odds ratio (AOR) of 2.53. Thus, the final sample size was 392. A sample size of 692 obtained from the first specific objective was used because it was the largest sample size estimated and would be sufficient for the study.

### Sampling technique

A two-stage stratified sampling technique (i.e. considering each Kebele as stratum) was used to select a representative sample size. In the first stage, we have used simple random sampling technique to select a representative Kebeles from the Hawassa city. In the second stage, we used a systematic random sampling technique to select the study households. In Hawassa city, there are 32 Kebeles and from all 32 Kebeles, nine Kebeles were selected by using a simple random sampling technique. Households with reproductive age group women who were non-pregnant were identified by the house-to-house census and a sampling frame was prepared. Finally, study participants were selected using a systematic sampling method. The first woman was identified by using simple random sampling and then after, at regular intervals, another woman was selected until needed sample size reached. If woman was absent from the household for 3 consecutive visits and there were no other options, the next nearest woman was included. One woman was included by using simple random sampling when two or more women existed in the selected households. To select discussant women a purposive sampling technique was used by considering variation in age, socio-economic and education status. All women included in the FGD were not participants of the quantitative study.

### Study variables

The outcome variable was utilization of long acting reversible contraceptive methods. The independent variables were socio-demographic variables such as age, religion, ethnicity, family size, wealth index, maternal/paternal education and occupation, marital status, number and sex of living children, and media availability; reproductive factors such as history of abortion, experience of child death, need of more children; factors related to communication such as communication with husband and health professionals; health service availability and other factors like perception, knowledge and attitude.

### Study tools and data collection techniques

Data collection was administered by 8 clinical nurses. Two BSc nurses intensively supervised the data collection process. Qualitative data collected using focus group discussion (FGD) until information saturation was reached. Each FGD consisted of 8 participants and were selected purposively from one to five networks of reproductive age group women. FGD guideline was used to explore ideas of reproductive age group. It was triangulated with the quantitative method. Two experienced health officers were assigned for note taking and tape recording while the public health expert with MPH facilitated the FGD. Quantitative data were collected at their households while FGD were conducted at proximal community gathering area.

### Data quality control

Data were collected using structured and interviewer-administered questionnaire. Firstly, the questionnaire was prepared in English. Secondly, it was translated into Amharic language. Finally, it was retranslated back to English to keep its consistency. The comparison was done to assess the inconsistency and non-accuracy between the two versions of the questionnaire. It was pre-tested on 5% of sample and one FGD was conducted in Kebeles which were not included in the actual study area. Then, any inconsistency and non-accuracy was corrected accordingly. Training was given for data collectors and supervisors by the principal investigator for 2 days. The training was focused on the objective, methods and data collection process. In addition, regular checkup for completeness and consistency of the data were made on a daily basis.

### Operational definitions

**Long acting reversible contraceptive methods** are a modern contraceptive method which includes implant and Intra Uterine Contraceptive Device (IUCD) and provides uninterrupted protection to women for 3 to 12 years.

**Short acting reversible contraceptive methods** are a modern contraceptive methods which are used in short time intervals from single use (e.g. the condom), daily intake (e.g. the pill) to up to 3 monthly application (e.g. injectable contraceptives).

**Knowledge** was measured using the 7 knowledge assessment questions. Knowledge questions were scored and pulled together and the mean score was computed to determine the overall knowledge of the participants. Based on that good level of knowledge assigned when the respondent answered the sum score of greater than or equal 75% of 7 knowledge assessment questions; moderate level of knowledge, when the respondent correctly answered between 50 and 74%; and low level of knowledge, when the respondent answered below 50% of 7 knowledge assessment questions.

**Attitude** was measured using the 8 attitude assessment questions. Accordingly, those who scored above mean, mean and below mean to the correct answers considered as positive and negative attitude, respectively.

### Data analysis

The data were entered into Epi Data 3.1 and exported to the Statistical Package for Social Sciences (SPSS) version 20.0 for analysis. All required variables recoding and computations were done prior to the main analysis. Descriptive analyses were conducted to obtain descriptive measures for the socio-demographic characteristics and other variables. Tables and graphs were used for the data presentation. Chi-square(X^2^) test was used to determine the overall association between explanatory and outcome variables. Cross tabulations was used to test the assumption of X^2^. A sensitivity analysis was conducted to investigate the effect of missing data by multiple imputations. Binary logistic regression was used to identify predictors of LARC methods utilization. The binary logistic regression analysis started with unadjusted analysis in which each potential predictor was assessed separately for its association with LARC methods utilization. Variables with *p*-values < 0.25 on the unadjusted analysis were entered into a multivariable logistic regression model to find out independent predictors of LARC methods utilization adjusting for other factors in the model. The variables were entered into the multivariable model using the backward stepwise regression approach. The main assumptions of logistic regression model (absence of outliers, multicollinearity and interaction among independent variables) were checked to be satisfied. Accordingly, none of the interaction terms was statistically significant indicating absence of a significant effect modification. Multicollinearity between the independent variables was also assessed using multiple linear regression. No evidence of multicollinearity was found as the variance inflation factor (VIF) for all variables was less than 10 and the tolerance statistic was greater than 0.1. The fitness of logistic regression model was also evaluated in model using the Hosmer-Lemeshow statistic and greater than 0.05. The presence and strength of association between LARC methods utilization and the predictors was assessed using adjusted odds ratios (AORs) with 95% CIs. Statistically significant association was declared when the 95% CI of the AOR did not include 1. Qualitative data were transcribed verbatim from the local languages into English language and presented in narratives using the respondents own words. The transcripts were read and checked two times by the professional transcribers for verification. The qualitative data were analyzed using a manual thematic analysis technique.

## Results

Socio-demographic characteristics of the study participants have been summarized in Table 1. From a total of 692, only 660 study participants answered questions, making a response rate of this study 95.3%. The mean (± standard deviation [SD]) of the age of participants was 25 (± 7) years. Majority of respondents were within the range of 20–35 years. The median family size of each household was 4 persons. According to this study, majority 387/660 (58.6%) of the study participants were from Sidama ethnic group. Majority 403/660 (61.1%) and 433/660 (65.6%) of the study participants were followers of protestant Christianity and married, respectively.

**Table 1:** Socio-demographic characteristics of study participants in Hawassa city, South Ethiopia, 2019 (*N* = 660)

Variable	Categories	number	Percent
Age	15–19	116	17.6
	20–36	481	72.9
	37–49	63	9.5
Marital status	Single	216	32.2
	Married	433	65.6
	Others	11	2.2
Religion	Protestant	403	61.1
	Orthodox	195	29.5
	Musilim	41	6.2
	Others	21	3.2
Ethnicity	Sidama	387	58.6
	Amhara	109	16.5
	Wolaita	64	9.7
	Others	100	15.2
Educational level	No formal education	44	6.4
	Primary level	235	34.2
	Secondary level	219	31.9
	Higher/university level	162	24.5
Occupation	Housewife	293	44.4
	Government employer	66	10.0
	Merchant	57	8.6
	Daily employer	244	37.0

**Fig. 1:**
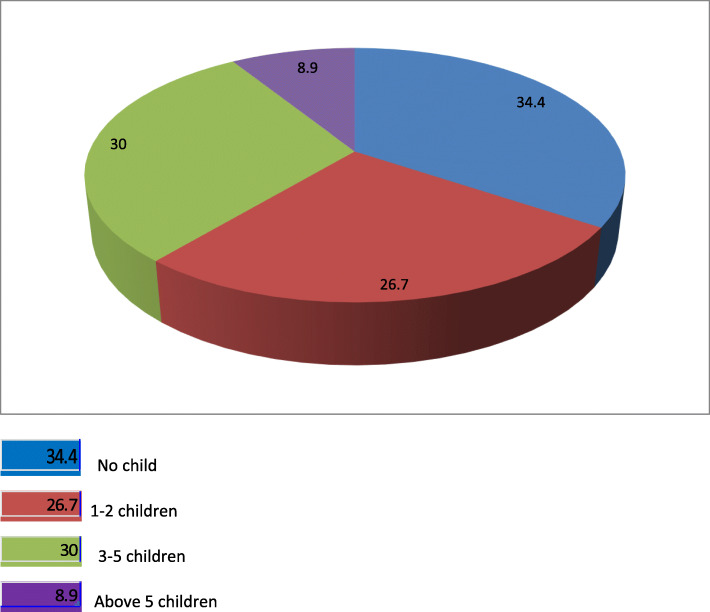
Number of children that reproductive age women have in Hawassa city, 2019

Two hundred twenty-seven (34.4%) of study participants had no child, 176(26.7%) had 1–2 children, 198 (30.0%) had 3–5 children and 59(8.9%) had greater than 5 children (Fig. 1).

### Average number of children that needed for one household from group discussants illustrated as follows

“*Large family size is not good. It is difficult to raise children and difficult to cover school expense. It is enough if you get four children on average. But no problems if you get more children as you have economy*”. (**G-1 D4 woman aged 32 years**)

*“Having four children, totally six family members are enough. Family size determined by household economy. What is the importance of having a large family size? It has no advantage; rather it is determined by economic status”.* (**G-2 D5 women aged 37 years**)

*“Four children two males and two females are enough. Having many children is not good because in order to raise properly and live quality life, having large number of children is not good”.* (**G-2 D2 women aged 28 years).**

*“People want to have 6 to 7 children but it is restricted by economy. Due to economic problems, saving and also preventing giving birth within short period of time is mandatory”*. (**G-3 D2 29 years-old woman**)

*‘At this time, having large family size or having many children is not good. A maximum of 6 children are enough. This (having limited number of children) is because, in order to raise children properly, and of economic problems. Previously, our mother gave up 12 children and at that time cost of living was not that much expensive and children were grown easily. Mothers are also not affected if she does not give many children and can raise her children in quality of life’*. (**G-3 D4 34 years-old woman**)

***“****Even I want to have up to 12 children. It is a gift of God and I don’t want to close my womb by my mouth. If God give me, I want to give birth but by interval and by raising my children properly. You know injection can’t prevent God’s will because for example, one of my friends was using implant but she became pregnant. That means implant cannot prevent God’s will. Having many children has its own advantages because while you give birth, you plan and adjust your budget for it since you are giving birth by spacing.”* (**G-3 D1 25 years-old woman**)

*“As discussant 1 said, no one hates children to have. Our mother said that ‘if you have many children, they will be grown differently and their fate also different’. For example if you have only two children, raising two of them may be simple. For example, we are 7 in number and each of us has different behavior and fate. If one becomes weak, the other becomes strong and if one has good husband and other may not have as a previous one does, so having many children provide many choices. But according to present situation and as science says, children should be grown to have quality life and you have to educate them properly and address them to be in a good position*”. (**G-3 D4 34 years-old woman**)

Only 242/660 (36.7%) of the study participants had good knowledge on long acting reversible contraceptive methods. Two hundred sixty-two (39.5%) of the study participants had positive attitude and the 399/660 (60.5%) had negative attitude on long acting reversible contraceptive methods (Table 2).

**Table 2:** Knowledge and attitude of the study participants towards long acting reversible contraceptive methods in Hawassa city, South Ethiopia 2019 (*N* = 660)

Variables	Categories	Number	percent
Knowledge	Good knowledge	242	36.6
	Moderate knowledge	141	21.4
	Poor knowledge	277	42.0
Attitude	Positive attitude	262	39.5
	Negative attitude	398	60.5

### Utilization of long acting reversible contraceptive methods

The overall utilization of long acting reversible contraceptive methods was 22% (95% CI = 19.5–25.5%) in the study area. Majority, 428/660 (64.9%) of the study participants had ever used contraceptive methods during their lifetime. Five hundred thirty-seven (81.3%) of women had discussed with husband on contraceptive methods choice (Table 3).

**Table 3:** Utilization of long acting reversible contraceptive methods among reproductive age women in Hawassa city, South Ethiopia, 2019 (*N* = 660)

Variables	Categories	Number	Percent
Ever used contraceptive methods	Yes	428	64.9
	No	232	35.1
Currently use contraceptive methods	Yes	357	54.1
	No	303	45.9
Currently using	Long acting Reversible	145	22.0
	Short acting	515	78.0
Communication with husband	Yes	537	81.3
	No	123	18.7

**Fig. 2:**
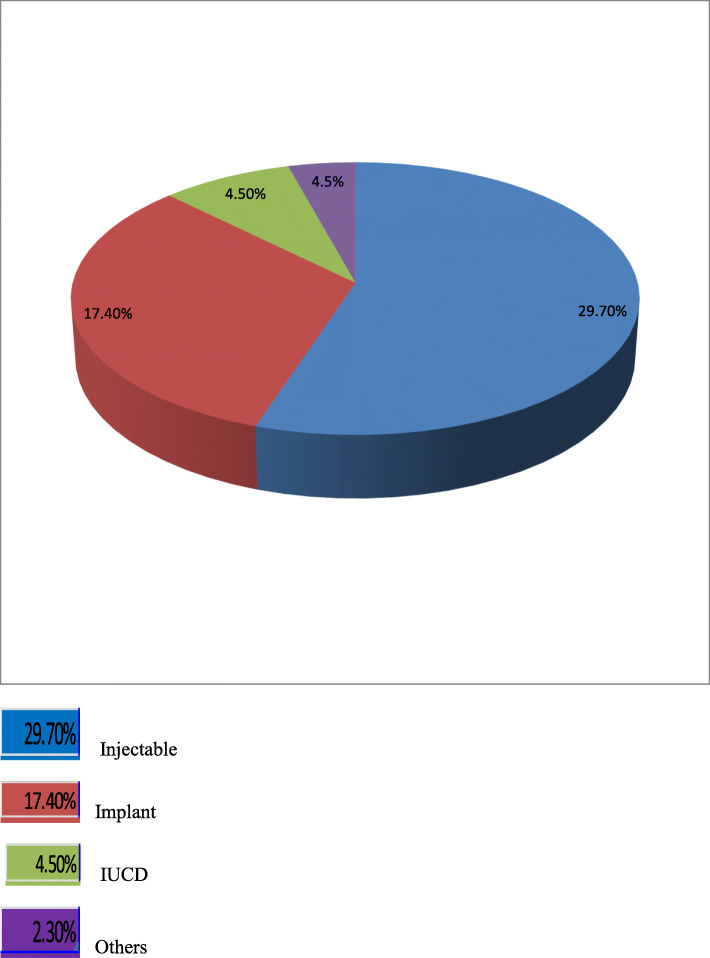
Current utilization of the modern contraceptive methods among reproductive age women in Hawassa city, 2019

Currently, 357/660 (54.1%) of the study participants were using modern contraceptive methods. The implant was the leading long acting reversible contraceptive method 115/660 (17.4%), followed by IUCD 30/660 (4.5%) (Fig. 2).

### Descriptive reasons for not utilizing long acting reversible contraceptive methods

**Fig. 3:**
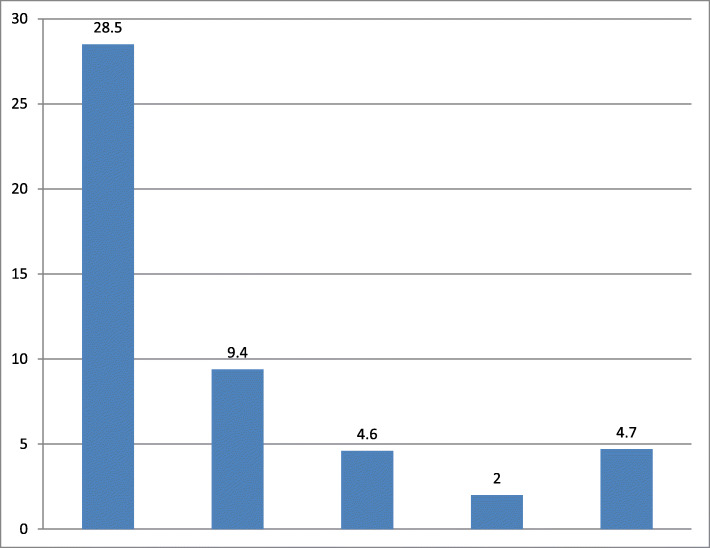
Descriptive reasons for non-utilization of the contraceptive methods among reproductive age group in Hawassa city, 2019

The main descriptive reasons that study participants did not use contraceptive methods were, 188/660 (28.5%) fear of side effects, 62/660 (9.4%) choose not to accept method at this time, 30/660 (4.6%) desire more children (Fig. 3).

#### Qualitative reasons for not utilizing long acting reversible contraceptive methods according to discussants expressed during group discussions are illustrated as follows

*“I am using injectable and one of my friends uses implant and it causes dizziness, weight loss or body decrement or thinness and when she went to a health facility, health professionals were not okay to remove it. So she went to a private facility and removed it by payment. Now she becomes healthy after removing it. So when I saw this, I don’t want to use implant. Also people said that injectable causes hypertension since it prevents menstruation, pills cause softening of uterus and causes cancer so it is difficult”*. (**G-1 D4 34 years-old woman**)

*“First, community members want to use it but after insertion some nurses were not okay to remove it if mother demanded to remove it. When I asked my friends “why they are not using long acting family planning” like five years implant, they said that “after insertion some health professionals refused to remove it”, and I think they were not using long acting reversible contraceptive due to that reason”*. (**G-3 D1 25 years-old woman**)

*“I have used implant and it has problem like wound, itching sensation and when I went to a health facility to remove it, they (health professionals) said that ‘for short period of time it may cause symptoms just like that and it will be resolved after your body adapt it’. When we wanted to remove it, they (health professionals) were not okay and simply they said that ‘if you have a problem, we will remove it’. Implant has so many problems like dizziness, itching sensation and irritability. What was a reason for this?”* (**G-3 D6 38 years-old woman)**

*“There were mothers who complained that “why does a pregnancy happen while we are using family planning methods?” Health professionals do not accepting mothers’ request to remove if mother demanded to remove it. They were not okay to remove if time were not reached as they said. My brother’s wife begun to have DM and HTN after using implant and when she went to remove implant, they were refused to remove and said that ‘implant inserted for three years, so its time is not reached’. Finally, they (health professionals) were removed implant when her husband fighting with them (health professionals) and she remained with these diseases”*. (**G-3 D7 25 Years-old Woman**)

### Predictors of long acting reversible contraceptive methods utilization

Results of the logistic regression analysis of LARC methods are shown in Table 4. Both bi-variable and multivariable analyses of predictors revealed that odds of LARC methods utilization were 4.0 times higher in women who have good knowledge as compared to those who have poor knowledge about LARC methods (AOR = 4.0; 95% CI = 1.66–9.60; *P* = 0.004). The odds of utilizing LARC methods increased 7.9 times for women who have a positive attitude (AOR = 7.9, 95% CI = 3.84–16.10) than women who have a negative attitude about LARC methods. The findings of FGD also support this. Focus group discussant said “*Some people believe that long acting contraceptives (Implant and IUCD) cause infertility if women used it for a long period of time. Even previously they believe that contraceptives cause infertility, but now they have started to use it after having awareness. Some people, who have no awareness and knowledge, by considering as it causes infertility, remove LARC but those who have knowledge about LARC using it properly”*. (**G-1 D1 27 years-old woman**) “*Community beliefs on LARC were, it causes infertility if they (women) stay for long period of time without giving birth. We know that within a community there are community members, who are not using family planning, and even they prefer to give birth until they have 6 to 7 children and some are using family planning accordingly, and their awareness is different”*. (**G-1 D4 34 years-old woman**).

**Table 4:** Bivariable and multivariable logistic regression analysis of predictorsof LARC methods utilization among reproductive age group in Hawassa city, South Ethiopia, 2019 (*N* = 660)

Variables	Bivariate analysis		Multivariate analysis
	Level	CI (95%)	CI (95%)
Knowledge	Good	19.00 (10.12–35.76)*	3.99 (1.66–9.60)**
	Moderate	3.80 (1.84–8.11)*	1.34 (0.50–3.60)
	Poor	1	1
Attitude	Positive	21.60 (12.56–37.22)*	7.90 (3.80–16.10)**
	Negative	1	1
Source of information	Health professionals	1	1
	Mass media	0.05 (0.02–0.15)	0.60 (0.13–2.83)
	Others	0.12 (0.04–0.33)*	0.43 (0.11–1.70)
Educational level	No formal education	1	1.
	Primary level	1.87 (0.24–14.61)	0.27 (0.09–8.65)
	Secondary	0.98 (0.21–4.52)	0.36 (0.03–4.72)
	Higher	6.80 (1.60–29.15) *	1.77 (0.13–23.31)
Discussion with husband	Yes	4.60 (2.20–9.60)*	2.70 (1.02–7.20)*
	No	1	
Ever use contraceptive	Yes	8.26 (4.40–15.30)*	2.30 (0.80–6.46)
	No	1	1
Number of children	0	1	1
	1–2	5.90 (2.88–12.38)*	0.56 (0.07–4.26)
	3–5	10.60 (5.26–21.35)*	0.86 (0.11–6.61)
	Above 5	25.70 (11.38–58.10)*	2.28 (0.25–20.61)
No desire to have children	Yes	2.53 (1.57–4.10)	8.20 (3.13–21.33)**
	No	1	1
Discussion with providers	Yes	4.90 (2.20–11.30)*	4.10 (1.36–12.55)**
	No	1	1

Note: 1 indicates the reference categories; a single asterisk (*) indicates a significant association (*p*-value < 0.05); double asterisk (**) indicates a highly significant association (*p*-value < 0.01)

In addition, discussion of women about LARC methods with health care provider (AOR = 4.1; 95% CI = 1.24–5.24) and husband (AOR = 2.7; 95% CI =1.02–7.20) were positively associated with utilization of LARC methods. Moreover, the odds of utilizing LARC methods increased 8 times for women who have no desire to have a child (AOR = 8.2, 95% CI = 3.13–21.30) as compared to those who have the desire to have a child.

## Discussion

A community-based cross-sectional study was conducted to assess utilization and predictors of LARC methods using mixed methods among reproductive age women in Hawassa city, South Ethiopia. The utilization of long acting reversible contraceptive methods among reproductive age women in Hawassa city was 22%. Good knowledge and positive attitude of women about LARC methods, no desire to have children, women discussion with health care providers and husbands had a significant association with utilization of the LARC methods.

In this study the utilization of the modern contraceptive and LARC methods among women of the reproductive age were 54.1 and 22%, respectively. Among this, majority of women (17.4%) utilized implant, followed by IUCD (4.5%). In contrary to the present finding, EDHS 2016 reported a lower utilization of modern contraceptives (36%) and implants (8%) [2]. This discrepancy might be due to the fact that EDHS survey included both rural and urban study participants. However, our study included study participants from urban area. In addition, a lower utilization of the modern contraceptive methods also reported from studies done in Arbaminch, Mekelle and Durame town, Ethiopia [12–14]. This difference might be due to the fact that difference in the sample size, study setting, and study period. However, the study conducted in Gedeo zone, south Ethiopia reported a higher utilization of modern contraceptive methods (74.8%), IUCD (6.6%) and implants (26.1%) than our study [15].

Multivariable analysis revealed that odds of LARC methods utilization were 4.0 times higher in women who have good knowledge as compared to those who have poor knowledge about LARC methods. This is consistent with the study findings from Arbaminch and Gedeo zone, Southern Ethiopia and Kicukiro town, Ruanda [15–17]. This might be attributed to the fact that women who have a good knowledge about LARC methods are more likely to outweigh the benefits and risks of using contraceptives. Moreover, it increases women’s capacity to make decision to utilize it.

The odds of utilizing LARC methods increased 7.9 times for women who had a positive attitude than women who had a negative attitude about LARC methods. This finding is in agreement with the studies done in Gesuba town, Gedeo zone, Addis Ababa city, Ethiopia and Calabar Metropolis, Southern Nigeria [15, 16, 18, 19]. This might be because of women who have a positive attitude about LARC methods are confident and more likely to minimize rumors of LARC methods. In addition, it increases women’s confidence to make decision about its utilization.

In addition, discussion of women about LARC methods with health care providers and husbands were positively associated with utilization of LARC methods. This finding is in agreement with studies conducted in North Shoa, Jigjiga Town, Ethiopia and Nepal [20–22]. This might be because women who had a discussion with health care providers and husbands were more likely to have information about LARC methods like duration of pregnancy prevention, effectiveness, method failure, and side effect. Also, these women were more confident to minimize rumors of LARC methods. Moreover, it increases women confidence to make decision about its utilization.

Moreover, the odds of utilizing LARC methods increased 8 times for women who had no desire to have a child as compared to those who had the desire to have a child. This finding agreed with studies conducted in North Shoa, West Harerge and Debre Markos town, North Ethiopia and Kucikiro district of Rwanda [17, 21, 23, 24].

### Limitation of the study

This study had a number of strengths. Among these, the community based nature and using both quantitative and qualitative methods provided representative and valuable information for all reproductive age group which is important to develop relevant policy strategy for efficient promotion of LARC methods utilization. Regardless of its strengths, our study has some basic limitations that might be considered while interpreting the findings. First, the cross-sectional nature of the study design does not exactly establish the cause and effect relationship. Secondly, the study might be prone to recall bias because of the information was collected by the study participants self-report.

## Conclusions

The utilization of LARC methods was far below the targeted health sector growth and transformation plan (22% v 40%) in the study area. The low utilization of LARC methods in the study area indicated that much work remains to be done to improve the health of women.

Good knowledge and positive attitude about LARC methods, women discussion with health care providers and husbands about LARC methods and no desire to have children were pertinent predictors of LARC methods utilization. Therefore, increasing knowledge and positive attitude of the women about LARC methods using various methods of health education should be considered. In addition, women’s discussion with health care providers and husbands about LARC methods to have an explicit understanding is also important. Moreover, providing relevant information about LARC methods for women who have no desire to have a child is also of paramount importance. Finally, further research using analytical study design to identify predictors of LARC methods utilization is required.

## Supplementary information

**Additional file 1.** The English version survey tools/questionnaire.

## Data Availability

We have sent all the available data and we do not want to share the raw data as we are doing related study.

## Abbreviations

AOR: Adjusted odds ratio
ANC: Antenatal Care
CI: Confidence Interval
DRC: Democratic Republic Congo
FGD: Focus group discussion
GTP 2: Growth and transformation plan 2
IRB: Institutional Review Board
IUCD: Intra-uterine contraceptive device
LARC: Long acting reversible contraceptive methods
OR: Odds ratio
PNC: Postnatal Care
SD: Standard Deviation
SPSS: Statistical packages for social science
WHO: World Health Organization
