# Supplementary material for: Utilization and predictors of long acting reversible contraceptive methods among reproductive age women in Hawassa city, South Ethiopia: a community based mixed methods
Source: Contracept Reprod Med. 2020 Jul 6;5:9. doi: 10.1186/s40834-020-00112-x (PMC7336663; doi:10.1186/s40834-020-00112-x)
Supplement: Supplementary file 1 — Additional file 1. The English version survey tools/questionnaire. [file 40834_2020_112_MOESM1_ESM.docx]

# ANNEX I. INFORMED CONSENT

**Introduction**

My name is ______________. I am working with Aklilu Tilahun who is doing a research for the partial fulfillment of Masters Degree in Public Health at HawassaUniversity. This questionnaire is intended to assess Prevalence and factors that affect utilization of long-acting reversible contraceptives methods among reproductive age group of married women in Hawassa city capital of SNNPR in Ethiopia. You are selected to be one of the Participants in the study.

**Purpose of this study:** the purpose of this research is to identify the factors affecting LARC utilization and its prevalence**.** The information that you provide here will be very helpful to the researcher to identify the factors and in designing priority intervention strategies for better implementation of LARC utilization**.**

**Benefits and Risks:** By participating in this study you will not receive any direct benefit. However, the information will help the researcher to understand factors affecting LARC among reproductive age group of married women to identify proper way for future interventions related to problem to be found. It will take you about 30 minutes and your participation will be volunteer based. In the mid of question if you are not comfortable to answer any/some question(s), you can leave it and no anybody will punish you. Your participation in this study will not have any risks.

**Confidentiality**: you will not be asked your name on to be written this questionnaire. All the information you give to us will be kept private. The information you give will kept in a locked file cabinet. Only the researcher will have access to see the answers you give. No information identifying you will ever be released to anyone outside of this data collection activity.

Address and Name of Principal Investigators Aklilu Tilahun

Phone number +251926095174

Email. Address aklilut18@gmail.com

Would you be willing to participate in the study?

If yes, I am in advance to ask you.

If no, please stop here

**Consent of the participant:**

I the undersigned have been informed that the purpose of this research project. Based on the above information I agree to participate in the research voluntarily.

Signature of Participant ----------------------------------Date-------------------------

# ANNEXES II. ENGLISH QUESTIONNAIRE

# Date of interview……………..

# Sub-city…………………………

# Interview number………………….

# Interviewee number………………….

# Interview starting time………………completed time……………….

# Supervisor name………………………date…………….signature…………

# Table4 .Questionnaires

| Code | Socio economic factors | | | | Skip |
| --- | --- | --- | --- | --- | --- |
|  | Question | Possible answer | | |  |
| 001 | What is your age? | ------ | | |  |
| 002 | Place of residence. | Urban…1 Rural…2 | | |  |
| 003 | What is your religion? | Protestant…..1Orthodox……………2  Musilim……3 Other specify................4 | | |  |
| 004 | What is you ethnicity? | Sidama……….1 Ahmhara………2  Wolaita……..3 Other specify…….4 | | |  |
| 005 | What is the highest level of school that you attend? | Illiterate……………..1  only read and write…2  1-8 grade……………3  9-12 grade…………..4  Above 12 grade……..5 | | |  |
| 006 | Your occupational status. | *Housewife………………..1*  *Government employer…2*  *Merchant………………..3*  *NGO employer…………4*  *Daily worker………….5*  *Other specify.................6* | | |  |
| 007 | What is your monthly income? | 200-500 Birr………1  501-1000Birr………2  1001-2000Birr……..3  2001-3000 Birr…….4  Above 3000 Birr……5. | | |  |
| 008 | Husband level of education | Unable to read and write..1Able to read and write…2Grade1-4…3 Grade5-8…4Grade9-12…5 Above 12 grades…6 | | |  |
| 009 | \| What is your marital status? \|  \| \| --- \| --- \| | Single……..1 Married…….2  Widowed……3 Divorced……4  Separated……5 specify other...6 | | |  |
| 010 | Have you history of sexual intercourse | Yes….1  No…..2 | | |  |
| 011 | Have you ever give birth before | Yes….1 No…..2 | | | 2skip201 |
| 012 | How many sons and daughters do you have? | Sons………..1 Daughters…..2 | | |  |
| 013 | Have you miscariege or died child | Yes…….1 No…….2 | | |  |
| 014 | If yes at what age | Before 7 months of pregnancy….1  After 7 months of pregnancy……2  With in one month of delivery…...3  After one month of delivery……..4 | | |  |
|  | Participants reproductive history | | | | |
| 201 | Have you ever heard about modern contraceptive methods? | | Yes…1 No…2 |  | |
| 202 | If yesQ201, what type of modern contraceptive do you heard? More than one answer is possible | | Pills……….1 Injectable…2 Implant……3 IUCD………4  Condom…….5 I don’t know…6. |  | |
| 203 | What are you sources of information? | | Neighbors /relatives… 1  Husband………………2  Health professional…..3  Mass media……………4  Other specify..................5 |  | |
| 204 | Do you have information about LARC methods? | | Yes…………………..1  No……………………2 |  | |
| 205 | If yes Q204, which methods? | | Implants………………..1  IUCD…………………….2  Other specify……………3 |  | |
| 206 | Are you currently using any contraception? | | Yes………...1  No…………..2 | skip to208 | |
| 207 | If Yes Q206, which method(s)? | | IUD…………..1 Implants………2  Pills…………..3 Injectable……..4  Condoms……….5  Other specify…...6 |  | |
| 208 | Did the provider ask if you were having a problem with the method (Probe: or did you mention aproblem)? | | Yes…1  No…2 | To Q14. | |
| 209 | Have you had a problem with your method (Probe: that you wanted to discuss with your provider). | | Yes…1  No…2 | skip to 213 | |
| 210. | Did the provider try to understand the nature of your problem? | | Yes…1 No…2 |  | |
| 211. | Did the provider suggest what you should do to resolve the Problem? | | Yes…1 No…2 |  | |
| 212. | Were you satisfied with the advice or treatment that you received for your Problem? | | Yes…1 No…2 |  | |
| 213. | Were you went health facility to obtain a specific contraceptive method? | | Yes…1  No…2 | skip to Q15 | |
| 214. | If yes for Q213, which method did you wants when you went to health facilities? (PROBE: Before your Consultation, did you have a specific method in mind?) | | Pill…1 IUD…2  Implant…3 Injectable…4  Others pecify……………5. |  | |
| 215. | Were you told when to return for a follow-up visit? | | Yes…1 No…2 |  | |
| 216. | Do you feel the information given to you during your visit was too little, too much, or just about right? | | Too little…1 About right…2  Too much…3 Don’t know…4 |  | |
| 217. | When meeting with the provider during your visit, do you think other clients could hear what you said? | | Yes…1 No…2  Not sure…3 |  | |
| 218. | Do you believe that the information that you shared about yourself with the provider will be kept confidential? | | Yes…1 No…2  Don’t know…..3 |  | |
| 219. | During your visit to the facility, how were you treated by the provider? | | Very well…1 Well…2  Not very well/ poorly…3. |  | |
| 220. | About how long did you wait between the time you first arrived at the facility and the time you saw a staff person for a family planning consultation? | | <15minutes…1  16-30 minutes…2  31-45 minutes 3  46-60 minutes…4  61 90minutes 5  >90 minutes…6  Don’t know…7. |  | |
| 221 | Do you feel that your waiting time was reasonable or too long? | | No waiting time Reasonable/ short…1Too long…2Don’t know…3. |  | |
| 222. | During your talk with the provider have you discussed STD/AIDS | | Yes…1  No…2 |  | |
| 223. | Did the provider encourage you to use condoms at the same time (Probe: simultaneously) as the family planning method you chose or are currently using? | | Yes…1  No…2 |  | |
| 224. | If no Q6, what is your reason? Mark most important reason only | | Chose not to accept a method at this time…1 Preferred method was not appropriate…2  Not available at all…3  No appropriate provider available that day…4  Not available at clinic…5  Fear of side effects…6  other ……………………………….7 |  | |
| 225 | Do you want to have any more children | | Yes……1 No……..2 |  | |
| 226. | How long would you like to wait from now before the birth of (a/another) child? | | Less than two year...1  More than two years…2  Don't know…3 |  | |
| 227. | Did the provider talk about having another child in the future? | | Yes…1 No…2 |  | |
| 228 | Have you discussed with your partner about which method to use? | | Yes…1 No…2 |  | |
| 229 | If yes, what was your partner opinion? | | Force you to choice a method what he want…1 Decide together…2  Prevent her from using any methods…3  Other specify............................................4 |  | |
| 230 | Which methods did the provider discuss with you? | | Pill…1 IUD…2Implant…3 Inject able…4 Other specify....................5 |  | |
| 231 | Have you ever used contraception before? | | Yes…1 No…2 |  | |
| 232 | If Yes, which method (s)? | | Pill…1 IUD…2Implant…3 Inject able…4Other specify........................5 |  | |
| 233 | What is your current reproductive goal for seeking contraception? | | Child spacing…1  Permanent limitation of family size…2  Other Specify...3 |  | |
| Part 3 | Factors related to knowledge and attitude | |  |  | |
| 301. | Which methods do you know from long acting methods? | | IUD…1 Implants…2Other specify......................3 |  | |
| 302 | Can implants prevent STI? | | Yes…1 No…2 Not sure…3 |  | |
| 303 | Implant can prevent pregnancies from 3 -5 years? | | Yes…1 No…2 Not sure…3 |  | |
| 304 | Implants are immediately reversible | | Yes…1 No…2 Not sure…3 |  | |
| 305 | IUCD can prevent pregnancy for ten to twelve years. | | Yes…1 No…2 Not sure…3 |  | |
| 306 | IUCD can prevent sexual transmitted disease. | | Yes…1 No…2 Not sure…3 |  | |
| 307 | IUCD is immediately reversible. | | Yes…1 No…2 Not sure…3 |  | |
|  | Attitude related | |  |  | |
| 308 | Using implant causes irregular bleeding? | | Agree…1 Disagree…2 Not sure…3 |  | |
| 309 | Insertion and removal of implant is highly painful? | | Agree…1 Disagree…2 Not sure…3 |  | |
| 310 | Implant cannot interfere routine activities. | | Agree…1 Disagree…2 Not sure…3 |  | |
| 311 | Implants do not move through the body after insertion. | | Agree…1 Disagree…2 Not sure…3 |  | |
| 312 | IUCD insertion doesn’t lead to lose privacy. | | Agree…1 Disagree…2 Not sure…3 |  | |
| 313 | IUCD doesn’t move through body after insertion. | | Agree…1 Disagree…2 Not sure…3 |  | |
| 314 | IUCD has no interference with sexual intercourse desire. | | Agree…1 Disagree…2 Not sure…3 |  | |
| 315 | IUCD doesn’t restrict normal activities. | | Agree…1 Disagree…2 Not sure…3 |  | |
|  | FGD guide questionnaire | | |  | |
| 1. | How is your understanding concerning modern contraceptives? What else? | | |  | |
| 2. | What do you know about long acting reversible contraceptive methods? (Probe; Implant and IUCD contraception? | | |  | |
| 3. | What are the advantages/disadvantages of LARC over short acting methods? What else? | | |  | |
| 4 | Would you like to know more about LARC? Why? | | |  | |
| 5 | When should people start to use LARC? Why? | | |  | |
| 6 | What are myths and beliefs with in community about LARC? | | |  | |
| 7 | What is the optimum family size you think is enough? Is having large family size useful or harmful? Why? | | |  | |
| 8 | Who should decide about family size? Why do you think so? | | |  | |
| 9 | Who should use LARC? Why? | | |  | |
| 10 | What kind of problems or complaints raised from users on health professionals while they are providing family planning services specially LARC? | | |  | |
| 11 | Is there any additional idea that you want to add on our discussion on LARC methods and related issues. | | |  | |

Thank You!!!
